# Comprehensive systems biology analysis of a 7-month cigarette smoke inhalation study in C57BL/6 mice

**DOI:** 10.1038/sdata.2015.77

**Published:** 2016-01-05

**Authors:** Sam Ansari, Karine Baumer, Stéphanie Boué, Sophie Dijon, Remi Dulize, Kim Ekroos, Ashraf Elamin, Clement Foong, Emmanuel Guedj, Julia Hoeng, Nikolai V. Ivanov, Subash Krishnan, Patrice Leroy, Florian Martin, Celine Merg, Michael J. Peck, Manuel C. Peitsch, Blaine Phillips, Walter K. Schlage, Thomas Schneider, Marja Talikka, Bjoern Titz, Patrick Vanscheeuwijck, Emilija Veljkovic, Terhi Vihervaara, Gregory Vuillaume, Ching Qing Woon

**Affiliations:** 1 Philip Morris International R&D, Philip Morris Products S. A., Quai Jeanrenaud 5, Neuchâtel 2000, Switzerland; 2 Zora Biosciences Oy, Biologinkuja 1, Espoo 02150, Finland; 3 Philip Morris International Research Laboratories, 50 Science Park Road, Science Park II, Singapore, Singapore 117406; 4 Biology consultant, Max-Baermann-Str. 21, 51429 Bergisch Gladbach, Germany

**Keywords:** Respiratory system models, Microarray analysis, Systems biology, Lipidomics, Proteomic analysis

## Abstract

Smoking of combustible cigarettes has a major impact on human health. Using a systems toxicology approach in a model of chronic obstructive pulmonary disease (C57BL/6 mice), we assessed the health consequences in mice of an aerosol derived from a prototype modified risk tobacco product (pMRTP) as compared to conventional cigarettes. We investigated physiological and histological endpoints in parallel with transcriptomics, lipidomics, and proteomics profiles in mice exposed to a reference cigarette (3R4F) smoke or a pMRTP aerosol for up to 7 months. We also included a cessation group and a switching-to-pMRTP group (after 2 months of 3R4F exposure) in addition to the control (fresh air-exposed) group, to understand the potential risk reduction of switching to pMRTP compared with continuous 3R4F exposure and cessation. The present manuscript describes the study design, setup, and implementation, as well as the generation, processing, and quality control analysis of the toxicology and ‘omics’ datasets that are accessible in public repositories for further analyses.

## Background & Summary

Chronic obstructive pulmonary disease (COPD) is a major cause of chronic morbidity and mortality worldwide^1^ and is often caused by the smoking of cigarettes. Biological processes involved in COPD development can be studied, at least in part, using rodent models of cigarette smoke (CS) exposure. C57BL/6 mice have a moderate deficiency of the serine protease inhibitor Serpina-1, which is more pronounced in females^2^, and develop emphysema and goblet cell metaplasia upon smoke exposure^2^. They are susceptible to smoke-induced airway remodeling, and are therefore a useful model with which to study emphysema initiation and progression^3^, which is characterized by reduced lung function, abnormal airway inflammatory responses, small airway remodeling, and the destruction of lung alveolar tissue common to early stages and aspects of human COPD^4,5^.

To investigate the impact of tobacco aerosol inhalation on the development of emphysema/COPD, we evaluated C57BL/6 mice subjected to exposure to the smoke/aerosol of two tobacco products, a conventional reference cigarette (3R4F) and a prototypic modified risk tobacco product (pMRTP), in a 7-month inhalation study. Female mice were randomly allocated to the following five groups: (1) Sham, exposed to filtered, conditioned air, (2) 3R4F, exposed to mainstream smoke from 3R4F cigarettes (750 mg total particulate matter (TPM) /m^3^ target dose), (3) pMRTP, exposed to a MRTP prototype aerosol (nicotine level in test atmosphere matched to that of the 3R4F group, i.e., 34 mg/m^3^), (4) Cessation, exposed to 3R4F for 2 months and then to filtered, conditioned air for up to 5 months, and (5) Switch to pMRTP, exposed to 3R4F for 2 months and then to pMRTP aerosol for up to 5 months. The animals were exposed for 4 h/day, 5 days/week, for up to 7 months. The test atmospheres in the animal breathing zone were regularly analyzed to ensure the quality and reproducibility of the exposure throughout the study period. Biomonitoring of the animals (Table 1) demonstrated that the aerosols were effectively taken up and in-life observations confirmed that the exposure concentrations of both 3R4F and pMRTP were well tolerated by the animals.

**Figure 1:**
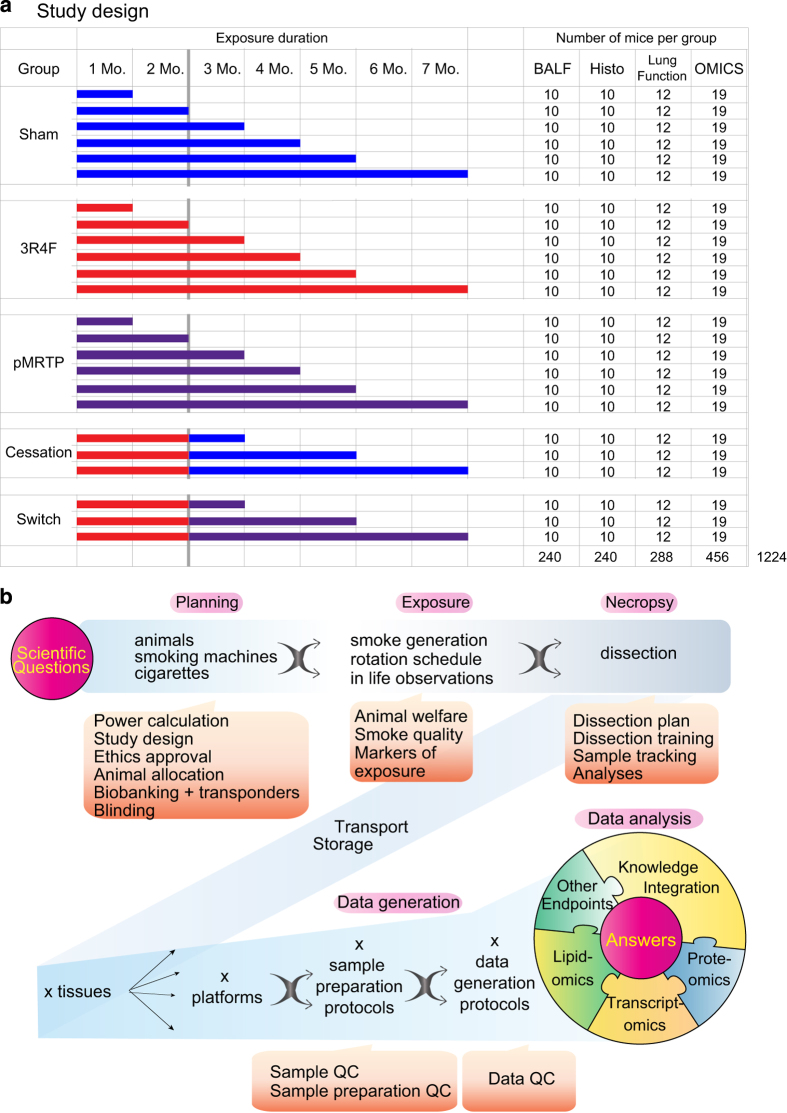
Experimental design. The study design entailed 5 different exposure groups, 6 necropsy time points, and 5 main endpoint categories, where mice were allocated for each category. The number of mice planned in the study per exposure group for each endpoint and time point and the design are summarized in (**a**). To ascertain the best quality of the data to answer the scientific questions, a number of quality control and randomization steps were put in place (**b**).

**Figure 2:**
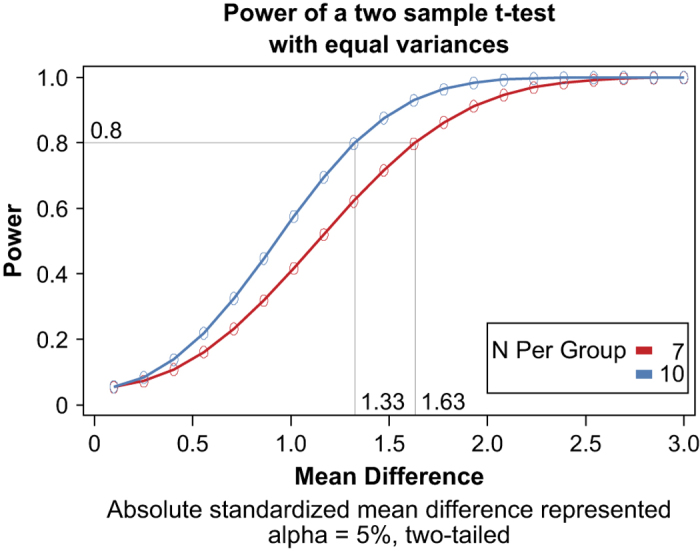
Power analysis. The plot shows the power of a two sample *t*-test given the mean difference between the two groups, for different number of replicates per group.

Pulmonary pathological and functional changes were assessed in dedicated groups of animals by standard histopathology and morphometric analyses, as well as by lung function testing after 1, 2, 3, 4, 5, and 7 months of exposure (Fig. 1). Pulmonary inflammation was further quantified by analyzing bronchoalveolar fluid (BALF) for inflammatory cell and mediator contents. Tissues and organs were collected (after 1, 2, 3, 5, and 7 months) for ‘omics’ analysis, according to a predefined dissection plan that ensured the best possible quality of the samples. Lung transcriptomics, proteomics and lipidomics profiles, respiratory nasal epithelium transcriptomics profiles, liver transcriptomics and lipidomics profiles, and blood transcriptomics and lipidomics profiles were obtained. In this systems toxicology approach, biochemical and transcriptional data provided a supportive mechanistic layer to the traditional toxicology endpoints investigated in parallel, while the quality control measures ensured that high quality and reproducible data were generated (Fig. 2).

Previously, we have published another *in vivo* study in rats exposed to a similar pMRTP^6^. We have also published a manuscript in *Food and Chemical Toxicology* that focuses on the interpretation of the data from this study as well as elucidating the biological impact of pMRTP exposure compared with 3R4F exposure^7^. The value of the present manuscript is the detailed description of the unique design and implementation of the complex inhalation study with many time points and exposure groups including sham, pMRTP, 3R4F, cessation, and switching groups. The generation, processing, quality control, and the accessibility of the data from several endpoints, such as physiological, disease related, molecular (e.g., gene expression, lipids, proteins), functional, and histopathological measurements are also described in detail.

This data descriptor article makes no claims regarding the toxicity or health consequences of modified risk tobacco products, and asserts no particular interpretation of these data.

## Methods

### Study design

The study design included five groups of female C57BL/6 mice: (1) Sham (fresh air-exposed), (2) 3R4F (exposed to mainstream smoke from 3R4F), (3) pMRTP (exposed to mainstream smoke from a prototype MRTP), (4) Smoking cessation (exposed for 2 months to CS and then to fresh air), and (5) Switching to pMRTP (exposed for 2 months to CS and then to pMRTP) (Fig. 1). The time point for switching was chosen based on the results of a previously conducted inhalation study on C57BL/6 mice indicating that lung inflammation, emphysema, and changed pulmonary function were apparent after 2 months of exposure to 3R4F^8^. Aerosol characterization for the pMRTP and 3R4F cigarettes, demonstrating reduction of a number of harmful and potentially harmful constituents is given in ‘Smoke chemistry’ file [Data Citation 1]. The allocation of animals to each endpoint was performed to optimize data quality while keeping the number of mice as low as possible, e.g., blood was collected from all groups to allow for all analyses to be done. An overview of all measurements shared in this manuscript can be found in the ‘Study overview file’ [Data Citation 1].

### Power calculation

Typically, 10 animals were used per treatment, exposure duration, and endpoint type because this group size has previously been shown to be reasonable for statistical interpretation of data^8–10^ while keeping the animal numbers in line with the expectations of our animal welfare policy. Figure 2 illustrates the power obtained for various sample sizes (*n*=7 and 10 per group) as a function of the standardized mean difference by performing a two-tailed two-sample *t*-test with equal variances and α=5%, the standardized mean difference being defined as the absolute difference between the means of the two groups compared divided by their common standard deviation. As shown, it is possible to detect with a relatively high power (0.8) standardized mean difference of ~1.3 with a sample size of 10. In case of a loss of 3 animals or samples, a reduced sample size of 7 still allows detecting standardized mean differences of ~1.6 with that power of 0.8.

### Ethics approval

All procedures involving animals were performed in an accredited, Agri-Food & Veterinary Authority (AVA) test facility in Singapore with approval of an Institutional Animal Care and Use Committee (IACUC), in compliance with the National Advisory Committee for Laboratory Animal Research (NACLAR) Guidelines on the Care and Use of Animals for Scientific Purposes^11^. Every animal related procedural change during the course of the study was communicated, discussed and approved by the IACUC committee, Corporate approval to conduct this study was provided by the PMI Animal Welfare Committee. In addition, our facilities are accredited by the Association for Assessment and Accreditation of Laboratory Animal Care (AAALAC).

### Animals

Female C57BL/6 mice bred under specific pathogen-free conditions were obtained from Charles River Laboratories (Wilmington, MA, USA) and were 8–10 weeks old (16.7–22.7 grams) at exposure initiation. Health certificates for all animals were provided by the supplier, animals were thoroughly inspected upon arrival to the facility, and serology, bacteriology, and parasitology analyses were performed on six mice prior to the start of the study and in the last month of the study (Harlan, Blackthorn, UK). Additionally, the serum of six reserve animals was analyzed for common infectious agents in months 3 and 7 (Health Screening Type SM246, Harlan). Metadata associated to all animals in the study can be found in the ‘Body weight ISA file (Metadata)’ file [Data Citation 1].

### Housing and husbandry

Mice were kept and exposed in a restricted-access animal laboratory under specific hygienic conditions. A maximum of eight mice were housed per cage (Cat. 1290D00SU, 454×266×155 mm, floor area 820 cm^2^, Techniplast, Varese, Italy). A cage enrichment (igloo, Biosys Corp Pte. Ltd., Singapore) was provided for each cage outside of the exposure period. Washed cages and bedding material (Lignocel BK 8–15, J. Rettenmaier & Sohne, GmbH & Co KG., Rosenberg, Germany) were autoclaved and changed twice weekly.

Tap water (filtered sequentially through 0.45 and 0.2 μm pore size filters) from water bottles with steam-sterilized sipper tubes was supplied ad libitum for each cage. A gamma-irradiated pellet diet (T2914C irradiated rodent diet, Harlan) was provided ad libitum via the cage lids and exchanged on a weekly basis. Food and cage enrichment were removed during exposure, but drinking water bottles were retained to allow animals free access to water.

The animal holding and procedure rooms were maintained at 21.8±0.4 °C and 21.6±0.3 °C and relative humidity of 55.5±0.7% and 51.0±1.2%, respectively (mean±s.d.). The light/dark cycle was 12 h/12 h. Exposure was carried out during the light phase. The general condition and health of the mice following smoke exposure were monitored by trained technicians and when needed by the attending veterinarian throughout the study.

### Moribund animals

Throughout the study, the animals were regularly monitored for overall health through a systematic health check schedule. In total, 11 animals were found dead or euthanized in the course of the study. All of them were replaced with weight-matched reserve animals, also undergoing the same exposure regime. Of the 11 animals, 2 were technical deaths (injured due to handling or sample collection), 2 were found dead, with no obvious cause, 2 were found dead with cause identified (ovarian cyst in both cases), 1 case of moribund with hydronephrosis identified, and 4 moribund animals with no obvious cause identified at post-mortem. The criterion for exclusion in these cases, and in general through the study was based on a series of humane endpoints, including rapid body weight loss (>20% in 1 week), weakness, inactivity and/or hunched posture. Details about moribund animals is given in the ‘Moribund and dead animals and missing values’ file [Data Citation 1].

### Allocation

Upon arrival, all animals were housed with minimal procedures for 2 weeks (implantation of transponders was performed in this period). Typically, any animal showing any findings (excessive alopecia, or signs of distress or disease) on arrival or during this acclimatization phase would be excluded from the allocation process. A total of 1,346 animals arrived to the facility. This included the planned 1,224 study animals, as well as approximately 10% reserve animals (122 animals). On arrival of the 1,346 animals, 1 animal was moribund (and was euthanized) and 50 mice arrived in transport boxes which were wet and partially damaged, resulting in a total of 51 animals rejected from the study on arrival (The animals were used for training and development activities) leaving 1,291 animals that entered the allocation process.

The allocation was performed by the study director, and from the initial 1,291 mice, 1,224 were allocated to the study groups, 40 animals allocated to 5 exposed reserve groups, and 27 reserve animals, of which 14 were used for scheduled health screenings (before and after the study). The remaining 13 animals were kept as reserve animals until 2 weeks after exposure start (which is our permissible replacement period of exposed animals), after which they were transferred out of the study for training purposes (a single reserve animal from this group was used as a replacement). The animals were allocated to experimental groups using a body weight-based stratification calculated using a custom *in vivo* animal management software package, Labautomation. Following allocation, each animal was provided with an uncoded number, containing information about study group and gender, and a coded number, which is randomly assigned and independent of animal group (but does contain gender information). The cross reference listing is available to a limited group of personnel, including the study director and the dissection manager, who use the lists for scheduling animals for necropsy. The data provided describes the 1,224 study animals plus 10 replacement animals (total 1,234 animals, any gaps in the coded number sequence correspond to unused reserve animals). On completion of the study, any remaining reserve animals were used for additional training of personnel or for method development following approval from the requisite animal welfare committee (IACUC).

### Blinding of research personnel

Due to the nature of whole body exposure study designs, it was necessary for the technicians to transfer the animals to their specific exposure chambers on a daily basis, and also to perform frequent monitoring of the aerosol constituents within the chambers. Therefore it is not possible to blind the animal treatment technicians in the treatment teams during the in life stage. Therefore, uncoded animal numbers are used on the cage cards, and group information is made available to the treatment technicians. However, all in life samples collected by the animal treatment technicians (blood, urine) were collected in tubes labelled only with the coded animal numbers, so downstream bioanalytical technicians were blinded to the animal groups. Similarly, during necropsy, only the coded numbers were used for animal identification, and to identify all collected tissues from the animals. In this way, the necropsy technicians were blinded to the animal groups, and all evaluation downstream of tissue collection is performed in a blinded manner.

### Test items

3R4F cigarettes were purchased from the University of Kentucky (http://www.ca.uky.edu/refcig) as soft packs of 20 cigarettes (10 soft packs/carton, 25 cartons/box). All cigarettes were stored in original packaging under controlled room temperatures (2–10 °C) but uncontrolled humidity conditions. Prior to smoking, the cigarettes were conditioned according to ISO Standard 3402 (ref. 12) for 7–21 days at a temperature of 22.1±0.2 °C and 58.1±0.8% relative humidity.

The pMRTP uses an extruded carbon heat source with CuO. Once ignited, the heat source heats the tobacco without burning it. A proprietary design thermally connects the tobacco to the carbon heat source and provides an effective and controlled temperature transfer to generate a nicotine-containing aerosol^13,14^. Additional constituents of the aerosol are water, glycerin, and tobacco flavors, together with reduced concentrations of tobacco pyrolysis products relative to conventional CS. pMRTP sticks were produced by Philip Morris Products S.A., Neuchâtel, Switzerland in seven batches (all in 2012). Following production, aerosols generated from a random sample of sticks were evaluated for levels of nicotine, glycerin, carbon monoxide (CO), TPM, formaldehyde, acrolein, and isoprene to ensure that the levels were within predetermined specification ranges. Each batch was therefore accompanied with an individual batch release certificate. The sticks were packaged by weight, with approximately 4,000 sticks sealed in aluminum bags per box, and were stored between 2–10 °C, but with uncontrolled relative humidity. Prior to smoking, pMRTP sticks were conditioned according to ISO Standard 3402^12^ at 21.6±0.2 °C and 55.4±1.4% relative humidity.

### Smoke generation

Throughout the study, all 3R4F ‘Kentucky Reference’ cigarettes were smoked according to the Health Canada smoking protocol^15^, with puff duration: 2.0 s; puff volume: 55 ml; puff frequency: 30 s, blocked ventilation, on 30-port rotary smoking machines (15 ports used) equipped with a programmable dual syringe pump (PDSP) and an active sidestream exhaust (type PMRL-G, SM2000).

Mainstream cigarette smoke (CS) was diluted with filtered, conditioned fresh air to the target TPM concentration of 750 mg TPM /m^3^ with approximately 34.4 μg nicotine/l aerosol.

The pMRTP aerosol concentration was matched to the same nicotine level as used for 3R4F exposure, i.e., 34.4 μg nicotine/l aerosol. The smoking conditions for pMRTP were: 12 puffs per stick; puff volume: 55 ml, puff frequency: 30 s, using modified SM2000 machines equipped with PDSP. The pMRTP aerosol or 3R4F CS was conveyed via glass tubing from the smoking machines to the exposure chambers.

The total flow rate through the whole-body exposure chambers was adjusted to ≥80 l/min (the target nicotine concentration was achieved by adjusting the dilution air). For the sham group, mice were exposed to filtered, conditioned fresh air, with a similar flow rate as in the aerosol-exposed groups.

### Exposure

Whole-body exposure chambers were used to provide controlled and reproducible exposure conditions.

The inhalation phase of the study started on 12th March 2012 and ended on 28th October 2012. It began with a 16-day adaptation period: the target 3R4F TPM concentration was 200 mg/m^3^ on study days 1–4, 400 mg/m^3^ on study days 5, 8, and 9, and 600 mg/m^3^on study days 10–12. From study day 15 (no exposure on the weekend), the target concentration was 750 mg/m^3^. A similar adaptation period was used for pMRTP-exposed mice, whereby the concentration of the pMRTP aerosol was adapted to match the nicotine levels of the 3R4F aerosol. From then on, mice were exposed to fresh filtered air, or to CS or pMRTP aerosol at target concentrations of 34.4 μg nicotine/l for 4 h per day, 5 days per week. Animals were exposed to filtered fresh air for 30 min after completing the first hour of exposure, and for 60 min after completing the second and third hours of exposure (exposure blocks) to prevent an acute carbon monoxide toxicity (for 3R4F) and control carboxyhemoglobin (COHb) levels not to exceed 50%.

Mice scheduled for dissection were exposed to the same smoking regimen for at least 2 consecutive days before necropsy (including the weekend in cases where dissection was performed on a Monday or Tuesday).

The position of the cages in the exposure chamber was rotated in each column of the chamber from top to bottom twice weekly to minimize position effects.

### Body weight

Mice were weighed regularly prior to allocation. At the time point of allocation to groups, a body weight determination was performed and used for the allocation procedure, which ensures similar body weights between the groups and an even distribution of weights within the exposure groups. Thereafter body weights were taken twice a week along the whole course of the study on a calibrated and validated balance. At the time point of necropsy, mice were weighed to determine anesthetic dose to use (weight before anesthetic). In cases when a mouse would be weighed twice in the same day, the average weight was recorded. Data resulting from body weight measurements can be found in the ‘Body weight ISA file (Metadata)’ and ‘Body weight Raw Data files’ [Data Citation 1].

### Hematology

Blood was obtained from the retro-orbital sinus directly into EDTA-coated blood collection tubes. A minimum of 50 μl of whole blood was required for analysis. The samples were analyzed within 2 h of collection using the Sysmex XT 2000 i clinical blood analyzer. The instrument measured the following parameters: red blood cells (RBC) and platelets (PLT) by direct current detection technology (impedance method) with hydrodynamic focusing, hemoglobin (HGB) by the sodium lauryl sulphate hemoglobin method and hematocrit (HCT) by cumulative pulse height detection method. Moreover, white blood cells (differential and relative counts), and reticulocyte numbers were estimated using flow cytometry method with a semiconductor laser. From these parameters, mean cellular volume (MCV) was calculated for RBC and HCT, mean cellular hemoglobin (MCH) was calculated for RBC and HGB, and mean cellular hemoglobin concentrations (MCHC) were calculated for HGB and HCT. All samples were analyzed in duplicate, and the results were accepted only when the second measurements was within±10% of the first measurement; an additional set of measurements was taken when the acceptance criteria were not met. Data resulting from hematology measurements can be found in ‘Hematology ISA file (Metadata)’ and ‘Hematology Raw Data’ files [Data Citation 1].

### Carboxyhemoglobin (COHb)

Blood COHb was determined by spectrophotometric measurement of absorbance at several wavelengths of the mixed hemoglobins in hemolysate. This was carried out during months 3, 4, and 6 (see Table 2).

### Nicotine and cotinine in plasma

The plasma levels of nicotine and cotinine were determined using established LC-MS/MS methods (ABF GmbH, Munich, Germany) from blood samples taken at the 7-month dissection (see Table 2).

### Nicotine metabolites measurement

Urine samples were collected overnight after exposure and stored at −80 °C, then used for high-performance liquid chromatography (LC) analysis of nicotine metabolites trans-3′-hydroxycotinine, norcotinine, cotinine, nicotine-N′-oxide, and nornicotine three times per group after 1,3 diethyl-2-thiobarbituric acid derivatization as described^16^. Values for the latest time point measured (6 months) are given in Table 2.

### Necropsy

Full necropsy was performed without prior fasting the day after the last exposure according to previously described methods^17^.

At the time of allocation, animals were randomly placed into exposure groups, including sub-groups with defined necropsy time points (months 1, 2, 3, 4, 5, and 7) and types of endpoints, with the constraint of keeping similar body weight averages across groups. Thereafter, these groups remained unchanged throughout the course of the study.

**Figure 3:**
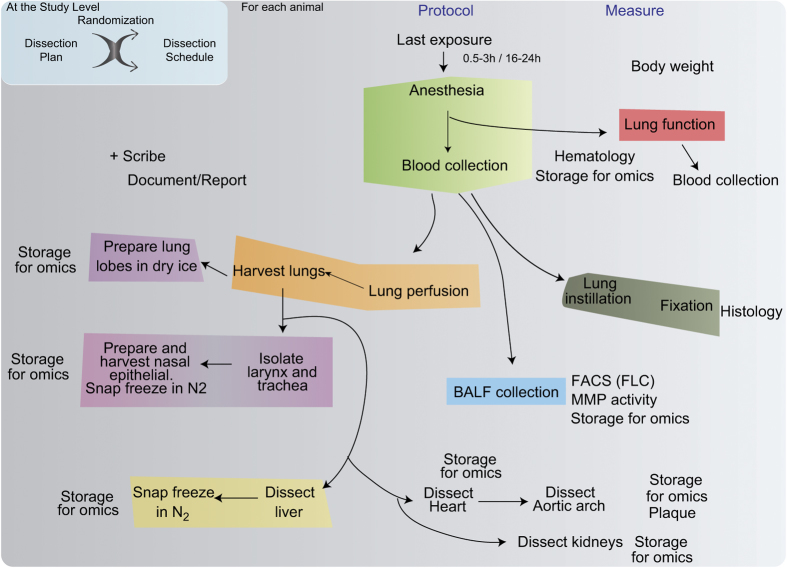
Dissection protocols set up to collect all endpoints with a minimum of animals while ensuring the best quality of the results.

Because the number of mice to be dissected at a single time point was large, the dissections spanned over a few days. Prior to each dissection, animals were randomized in the preparation of the dissection schedule. The dissection coordinator ensured that there was no bias in the spread of animals across the exposure groups for each dissection day and dissector. On the day of dissection, animals were dissected based on the dissection schedule. The original treatment group to which an animal belonged was not known by the person performing dissection. Different dissection protocols were used, depending on the endpoint in focus (Fig. 3).

### Dissection plan for histology-histomorphometry samples

Mice were anesthetized with an intraperitoneal injection of pentobarbital (100 mg/kg). Blood collection was performed via the retro-orbital sinus after the mice reached a deep plane of anesthesia. Two aliquots of 100 μl whole blood were collected in RNAprotect tubes (Qiagen, Hilden, Germany) for nucleic acid extraction. The blood was inverted at least 10 times and left to stand upright at room temperature overnight to ensure efficient cell lysis before storage at −80 °C. The remaining blood was collected in an EDTA-coated tube, inverted 10 times and left to stand for at least 30 min before storage at −80 °C. Complete exsanguination was performed by severing the aorta, then the mice were dissected to collect the lungs.

The respiratory tract organs were exposed by making an initial mid-ventral cut exposing the thorax regions. The rib-cage was opened by cutting adjacent to the sternum, exposing the lungs, trachea and larynx. Any connective tissue was carefully cut, enabling the removal of the lungs larynx and trachea from the thoracic cavity as a single unit. After removal, a small incision was made at the mid-point of the trachea between the larynx and the bifurcation. A tracheal cannula was then inserted into the incision and tied with a ligature. The cannula was connected via surgical tubing to fixative bottle. The lungs were instilled with the dorsal side facing down. Fixative (EGAFS; glycerin (5%), acetic acid (5%), formaldehyde (4%), NaCl (0.4%)) was instilled at a hydrostatic pressure equivalent of 15 cm water column. After instillation, the trachea was ligated, and the cannula removed. The lungs were then instilled in an ‘inflated’ manner for 48 h in EGAFS, followed by transfer to ethanol (70%) until processing for histology.

### Dissection plan for ‘omics’ samples

‘Omics’ tissue collection was conducted 0.5–3 h after the last exposure (Omics1) to include acute exposure effects or within 16–24 h after the last exposure (Omics2) to determine only the longer-lasting exposure effects. Mice were anesthetized with pentobarbital as described above. Complete exsanguination was not performed to facilitate pulmonary perfusion through the right ventricle using a 27-G needle with ice-cold 0.9% NaCl solution at a hydrostatic pressure of 100 cm to clear the lungs from remaining blood cells. Exit cuts were made at the right atrium and descending aorta for drainage. Complete perfusion of the liver and lungs occurred within 2 min and was indicated by the organs turning pale.

After perfusion, the heart was first collected, then the left and right lung lobes. This was followed by dissecting the entire liver and extraction of the nasal epithelium by two dissectors simultaneously. All tissues were collected within 15 min from the start of perfusion to avoid tissue degradation and to maintain good quality RNA. Organs were immediately snap-frozen in liquid nitrogen before storage at −80 °C. Efforts to minimize RNA degradation, such as the use of RNaseZAP (Life Technologies, Carlsbad, CA, USA) for cleaning of tools and surfaces, were strictly adhered to.

### Lung function measurement

Measurements were performed on animals from a dedicated group 18–24 h after the last exposure using unrestrained whole body plethysmography methodology (FlexiVent system, SCIREQ, Montreal, Canada) as described previously^18^. Mice were deeply anaesthetized by intraperitoneal (i.p.) injection of 50 mg/kg ketamine and 0.33 mg/kg medetomidine. Subsequently, mice were tracheotomized and their tracheas cannulated and connected to a computer-controlled small animal ventilator. Prior to the measurements, paralysis was induced by the i.p. administration of pancuronium bromide (0.8 mg) to avoid interference from spontaneous respiration followed by default ventilation (baseline, no maneuvers). Regular quasi-sinusoidal ventilation was delivered with a frequency of 150 breaths/min and a tidal volume of 10 ml/kg.

FlexiVent perturbation maneuvers were then carried out, including the maximal vital capacity maneuver (termed TLC by SCIREQ), quasi-static pressure-volume loops, single compartment (snapshot), constant phase model (primewave-3), and negative pressure forced expirations.

Data resulting from lung function measurements can be found in ‘Lung Function ISA file (Metadata)’ and ‘Lung function Raw Data’ files [Data Citation 1].

### Bronchoalveolar lavage fluid collection

**Figure 4:**
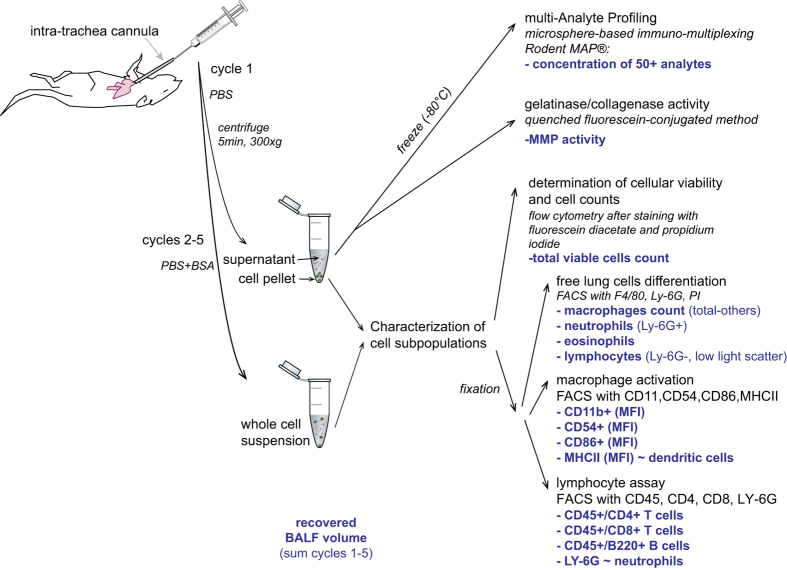
Methods used for bronchoalveolar lavage fluid (BALF) collection and characterization.

Mice were anesthetized with i.p. administration of 100 mg/kg pentobarbital. Whole blood was collected in lithium heparin tubes via the retro-orbital sinus after the mice had reached a deep plane of anesthesia. The tube was inverted at least 10 times and left to stand for at least 30 min before being centrifuged at 15000×g for at least 1.5 min to obtain a minimum of 50 μl of plasma, which was stored at −80 °C until processing for lipidomics assessment. Complete exsanguination was performed by severing the aorta. The rib cage was dissected away from the thoracic cavity to expose the lungs, which were cannulated *in situ* via the trachea with a 0.9 mm cannula. Lavage was performed using a 1 ml syringe with five consecutive cycles of filling and emptying with pre-warmed (37 °C) phosphate-buffered saline (PBS) for cycle 1 and PBS containing 0.325% bovine serum albumin for cycles 2–5. Each cycle involved filling and emptying with 1 ml of lavage medium, which was then repeated. The supernatant from cycle 1 was obtained by 300×g centrifugation for 5 min at 4 °C, then frozen in aliquots at −70 °C for multianalyte profiling (see ‘Bronchoalveolar Lavage Fluid (BALF) ISA file (Metadata)’ file [Data Citation 1]. Samples of bronchoalveolar lavage fluid (BALF) leukocytes (free lung cells, FLCs), contained in the cell pellet of cycle 1 combined with the complete cell suspension of cycles 2–5 were stored at 4 °C to characterize and enumerate cell subpopulations (Fig. 4).

### BALF-FLCs

BAL cells were analyzed as previously described^9^: aliquots were taken for FLC counting/viability determination and FLC differentiation/activation by flow cytometry (FACSCanto, BD Biosciences, San Jose, CA, USA). Cell numbers and viability were determined on fresh aliquots while differential counts (macrophages, neutrophils, lymphocytes, dendritic cells, and eosinophils) and activation markers were evaluated after formaldehyde fixation (2%, 18–24 h at 3–8 °C). Dendritic cell differentiation (high MHCII expression) and alveolar macrophage activation (CD11b, CD86, CD54, and MHCII) were measured as previously described^9^ (Fig. 4). For the FLC leukocyte differential count, the BALF cells were stained with an antibody master mix targeting the Ly6g and F4/80 antigens (Ly6g-FITC; F4/80-APC) and DNA content (PI).

The sub-differentiation of BALF lymphocytes into CD4+ T-cells, CD8+ T-cells, and B-cells was analyzed (Ly6g-PE, CD4-PerCP, CD8-PE-Cy7, B220-APC-Cy7, CD45-FITC, CD69-APC). Only measurements with >500 events in the lymphocyte gate were considered for a valid calculation of lymphocyte proportions. To check for non-specific staining, additional samples were stained with appropriate isotype control antibodies. Measurements were corrected for autofluorescence/non- specific fluorescence.

Data resulting from BALF FACS assessments can be found in ‘BALF FACS analyses reports’ and ‘BALF free lung cell count and enzymatic activity’ files [Data Citation 1].

### Proteolytic activity in BALF

Metalloproteinase (MMP) activity in BALF supernatants was determined using a commercially available assay kit based on the cleavage of fluorochrome-labelled gelatin by gelatinolytic MMPs in BALF (EnzChek Gelatinase/Collagenase Assay Kit; Molecular Probes, Eugene, OR, USA). After collection, BALF samples were stored at −70 °C until analysis, with no freeze-thaw cycles. Samples were analyzed within 3 months of collection (Fig. 4).

Data resulting from BALF proteolytic activity measurements can be found in ‘BALF free lung cell count and enzymatic activity’ file [Data Citation 1].

### Multianalyte profiling in BALF

Cell-free BALF supernatants were analyzed by Myriad RBM (Rules Based Medicine, Austin, TX) using a multiplexed bead array (RodentMAP v 2.0) (Fig. 4). Six sample shipments on dry ice were made, each corresponding to one of the six exposure periods (1, 2, 3, 4, 5, and 7 months of exposure), and six batches of measurements were performed over a total period of about 7 months. Therefore, the list of analytes measured changed over time (as the vendor changed the content of the RodentMAP), with three specific analytes measured only on the samples from the first four shipments, five specific analytes measured in samples from the last two shipments, and 55 other analytes on all samples. A total of 63 analytes generally considered biomarkers of inflammatory responses were determined.

Data resulting from BALF MAP assessments can be found in ‘BALF analytes’ file [Data Citation 1].

### Histology

The right and left lung lobes of 10 mice per exposure group and time point were paraffin-embedded. Left lung lobes were used for morphometric and histopathological evaluation, while right lung lobes were stored as reserve tissue. Step-serial sections (4 μm thick) were made from paraffin-embedded left lung lobes at intervals of 150 μm and stained with hematoxylin-eosin (Sigma), periodic acid-Schiff (PAS)-alcian blue (Merck), and resorcin-fuchsin (Ellipsiz). Histological slides were digitalized using an Aperio scanner (Aperio Technologies).

Five lung sections were selected for histopathological evaluation according to the following strategy:

- Section 1: taken at approximately 450*–*600 μm in front of section 2
- Section 2: taken at approximately 450*–*600 μm in front of section 3
- Section 3: section generated through the left main bronchus with branching secondary bronchi   visible
- Section 4: taken at approximately 450*–*600 μm behind section 3
- Section 5: taken at approximately 450*–*600 μm behind section 4

This approach provided central and peripheral aspects of the lung parenchyma for histopathological investigation.

The evaluation of goblet cells (main bronchus) and bronchus-associated lymphoid tissue (BALT) were performed on lung section 3 because the main bronchus was best represented at this level. XXL-PAS-positive macrophages, a type of pulmonary macrophage localized in the alveolar lumen, were also counted from section 3. Cellular sizes ranged from 18–28 μm. All other histopathological endpoints were evaluated separately for each lung section (five independent data sets).

Emphysema and fibrosis were evaluated histopathologically in a semi-quantitative manner (Histovia GmbH, Overath, Germany) using a severity score according to a defined grading system: 0=no different from normal morphology; 1=minimally different from normal morphology; 2=some areas minimally different and some areas moderately altered compared with normal morphology; 3=moderately altered compared with normal morphology (both local severity and spread distribution); 4=some areas moderately different and some areas severely altered compared with normal morphology; 5=overall severe alteration compared with normal morphology (both local severity and spread distribution). Note that, to the extent possible, the full severity scale was used, using reference slides for strong cigarette-smoke induced effect as a score 5. This means that the scores should be appreciated relatively within this particular study and that findings may be found with greater severity in other experimental setups.

Morphometrical assessment of pulmonary emphysema was performed on one central section (section 4) per mouse lung on digital images using Visiopharm Integrator System stereology software, version 4.2.9.0 (Horlsholm, Denmark). Morphometrical measurements of mean chord length (Lm) and destructive index (DI) were executed using approximately 30% of the total lung slide, which was randomly selected using an automated image capture procedure. For the measurement of bronchial attachments, 100% of the slide was evaluated.

The mean chord length was determined using three randomly orientated lines per image with a guard zone of 100 μm. The destructive index was determined using a grid structure of 4×5 measurement points per image. The number of bronchiolar attachments was determined on four counting frames per image, equivalent to 30% of the entire slide.

Annotation and data resulting from lung histopathology and histomorphometry assessments can be found in ‘Histology and histomorphometry ISA file (Metadata)’ and ‘Lung Histopathology and Histomorphometry Raw Data’ [Data Citation 1].

### Lung cryoslicing

Frozen left lungs were cut into sections (20 μm thick) with a cryostat (Leica CM3050 S), and 20 sections were collected in sterilized tubes filled with RLT (containing 1% 2-mercaptoethanol) lysis buffer (Qiagen) for RNA isolation. For proteomics analyses, frozen right lungs were cut into sections (40 μm thick) with the cryostat, transferred into empty tubes and stored at −80 °C until required for further processing. All samples were randomized and cryosliced in one batch.

### Omics analyses

**Figure 5:**
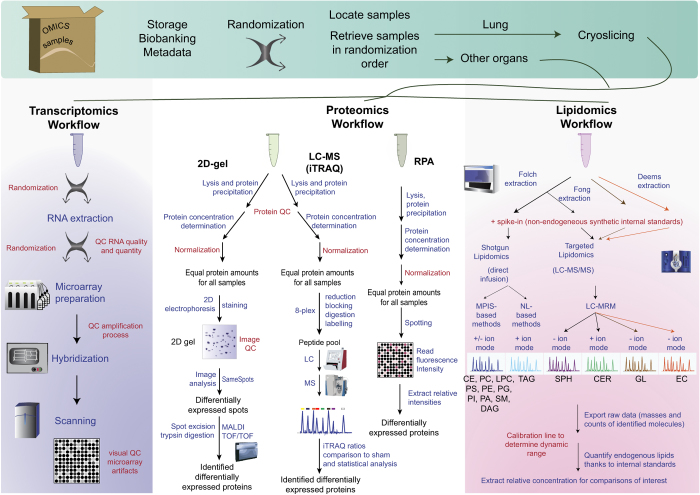
Methods used for ‘omics’ data generation.

Transcripts, proteins, and lipids levels were profiled in parallel from tissues obtained from ‘omics’ animals (Fig. 5).

### Transcriptomics

Total RNA was isolated from tissues (lung, nasal epithelium and liver) using the miRNeasy Mini Kit (Qiagen) and from blood using the RNeasy Protect Animal Blood kit (Qiagen), dissolved in an appropriate volume of RNAse-free water, quantified using the Nanodrop 1000 Spectrophotometer (Thermo Scientific, Waltham, MA, USA) and quality checked using the Agilent 2100 Bioanalyzer (Agilent Technologies). For blood samples, total RNA (80 ng) was reverse-transcribed into cDNA and amplified using the Ovation RNA Amplification System V2 (NuGEN, San Carlos, CA, USA). For tissue samples, 100 ng of total RNA was reverse-transcribed to cRNA using the Affymetrix® HT 3’ IVT express kit (Affymetrix, Santa Clara, CA, USA). cDNA/cRNA was then purified using magnetic beads to remove unincorporated nucleotide triphosphates, salts, enzymes, and inorganic phosphates. Purified cDNA/cRNA was quantified and labeled using an enzymatic attachment of nucleotides coupled to biotin. Fragmented, labeled cDNA (50 μl)/cRNA (33,3 μl) was added to 170/216,7 μl of Hybridization Cocktail Master Mix (Affymetrix, Santa Clara, CA), then denatured for 2 min (cDNA) or 5 min (cRNA) at 99 °C and 5 min at 45 °C, followed by centrifugation at Vmax for 1 min. A total of 200 μl of the cDNA/cRNA cocktail was then hybridized on MG430 2.0 GeneChips (Affymetrix). The arrays were incubated in the GeneChip Hybridization Oven 645 for 1 y±3 h at 45 °C with a rotation speed of 60 r.p.m. After hybridization, the arrays were washed and stained on a Fluidics Station FS450 using Affymetrix GeneChip Command Console Software (AGCC software version 3.2) with protocol FS450_0004 for Nugen Protocol (blood samples) and FS450_0001 for the IVT express protocol (tissue samples), then scanned using the GeneChip Scanner 3000 7G. Raw images from the scanner were saved as DAT files. Using the AGCC Viewer software application, each image was checked for artifacts, overall intensity distribution, checkerboards at the corners, a central cross to ensure adequate grid alignment, and readability of the array name. The AGCC software automatically gridded the DAT file image and extracted probe cell intensities into a CEL file. These files were further processed (MAS5.0) with Affymetrix Expression Console software (version Build 1.3.1.187) for a first quality check of the data.

Data resulting from transcriptomics analyses have been submitted to ArrayExpress [Data Citation 2].

### Lipidomics

Plasma was obtained as described earlier and stored at −80 °C prior to analysis. Lungs from ‘omics2’ mice were perfused through the right ventricle using a 27-G needle with ice-cold 0.9% physiological saline to remove contaminating blood cells. After dissecting the heart, the superior, middle, and post-caval lobes from the right lung were collected and snap-frozen immediately in liquid nitrogen before storing at −80 °C. Similarly, a piece of liver was frozen and stored in the same way.

Tissue samples were pulverized with a CP02 CryoPrep Dry Pulverization System (Covaris). Plasma samples were thawed slowly on ice. All samples were homogenized in ice-cold 70% methanol-H_2_O containing 0.1% butyl-hydroxy-toluene (BHT) at a concentration of 100 mg/ml. Homogenized samples were stored at −80 °C prior to lipid extraction and analysis. All lipidomics analyses were performed by Zora Biosciences Oy (Espoo, Finland).

#### Lipid extraction

Robotic assisted 96-well sample preparation and extraction was performed using a Hamilton Microlab Star system (Hamilton Robotics, Bonaduz, Switzerland). A modified Folch protocol, using chloroform, methanol and acetic acid for liquid-liquid extraction^19^, was applied to extract a broad lipid type spectrum^20,21^. This extraction procedure is efficient and robust over a wide lipid concentration range^22^. This method was used to extract glycerolipids, glycerophospholipids, sterol esters and sphingolipids, except for sphingosines and spingosine-1-phosphates which were extracted with 1.1 ml of ice-cold methanol containing 0.1% BHT. The Hamilton robot system was used to extract gangliosides using the methodology described by Fong and colleagues^23^ with minor modifications. Eicosanoids were extracted according to the procedure by Deems^24^. Prior to extraction, the samples were spiked with known amounts of internal standards (IS, see Table 3). This set of ISs were used to quantify the endogenous lipids in samples and controls as described below. Following lipid extraction, samples were dried under a gentle stream of nitrogen. Samples for shotgun lipidomics, sphingolipidomics and gangliosides were reconstituted in chloroform:methanol (1:2, v/v), whereas samples for sphingosine/sphingosine-1-phosphate and eicosanoids were reconstituted in methanol. The final extracts were stored at −20 °C upon mass spectrometry analysis.

#### Shotgun Lipidomics

Shotgun lipidomics was carried on a QTRAP 5500 (Sciex). Quantification of molecular glycerolipids, glycerophosopholipids and sterol esters was assessed by shotgun lipidomics as previously described^19^. Samples were loaded into 96-well plates (twin.tec PCR Plate 96, Eppendorf AG, Hamburg, Germany) and sealed with an aluminium foil (Heatsealing Foil, Eppendorf AG). Aliquots of 10 μl were aspirated and infused. Precursor ion and neutral loss scans were carried out in positive and negative ion modes, as described previously^25–27^. On the TriVersa NanoMate electrospray ionization (ESI) voltages applied were typically 1.3 kV and −1.3 kV in positive and negative ion modes respectively. The gas pressure was set typically to 0.75 psi in both polarity modes. In the positive ion mode the following MS settings were used: curtain gas; 20, collision gas; 6, interface heater; 60, declustering potential; 30, entrance potential 10 and collision cell exit potential; 20. In negative ion mode the following settings were used: curtain gas; 20, collision gas; 6, interface heater; 60, declustering potential; −100, entrance potential −10 and collision cell exit potential; −20. Q1 and Q3 quadrupoles were operated in unit resolution mode.

#### Sphingolipidomics

Molecular ceramides, glucosylceramides, lactosylceramides and globotriaosylceramides were analyzed as previously described^28^. Briefly, the individual species were separated using an Acquity BEH C18, 2.1×50 mm column with a particle size of 1.7 μm (Waters, Milford, MA, USA) assessed on a UHPLC system comprising of a CTC HTC PAL autosampler (CTC Analytics AG, Zwingen, Switzerland) and a Rheos Allegro pump (Flux Instruments, Reinach, Switzerland). A 25 min gradient using 10 mM ammonium acetate in water with 0.1% formic acid (mobile phase A) and 10 mM ammonium acetate in acetonitrile:2-propanol (4:3, v/v) containing 0.1% formic acid (mobile phase B) was used. The column oven temperature was set to 60 °C and a flow rate of 500 μl/min was used. A QTRAP 5500 mass spectrometer (Sciex, Concord, Canada) equipped with an electrospray ion source, was used for mass spectrometric determination. The instrument was operated in multiple reaction monitoring (MRM) mode in positive ion mode as previously described^28^. 78 MRM transitions were monitored using a dwell time of 20 ms. Q1 and Q3 quadrupoles were operated in unit resolution mode. The collision energy was set at 40 eV for ceramides, 45 eV for glucosyl- and lactosylceramides and 66 eV for globotriaosylceramides. Nitrogen was used as collision gas. The ESI voltage was set at 5000 V and the ion source temperature at 400C.

Sphingosines and sphingosine-1-phosphates were analyzed on a similar system as above. The individual species were separated using an AQUASIL C18, 2.1×50 mm column with a particle size of 5 μm (Thermo Fisher Scientific, San Jose, USA). A 19 min gradient using 5 mM ammonium acetate in ultra-pure water (UPW):methanol (1:1) with 0.1% formic acid (mobile phase A), 5 mM ammonium acetate in methanol with 0.1% formic acid (mobile phase B) and 10 mM ammonium acetate in isopropanol with 0.1% formic acid (mobile phase C) was used. The column oven temperature was set to 60 °C and a flow rate of 750 μl/min was used. For sphingosines and sphingosine-1-phosphates, the individual species were monitored in multiple reaction monitoring (MRM) mode in positive ion mode. 22 MRM transitions were monitored using a dwell time of 25 ms. Q1 and Q3 quadrupoles were operated in unit resolution mode. The collision energy was set at 22 eV for sphingosines and 21 eV for sphingosine-1-phosphates. Nitrogen was used as collision gas. The ESI voltage was set at 4500 V and the ion source temperature at 550 °C.

#### Ganglioside lipidomics

Gangliosides were analyzed as described previously^29^, except that 10 mM ammonium acetate and 0.1% formic acid were used in all solvents instead of ammonium formate. The analysis was assessed on a 6500 QTRAP (Sciex, Concord, Canada) equipped with a similar UHPLC system as described above. The individual species were separated using an Acquity BEH C18, 2.1×50 mm column with a particle size of 1.7 μm (Waters, Milford, MA, USA). A 32 min gradient using 10 mM ammonium acetate in methanol with 0.1% formic acid (mobile phase A), 10 mM ammonium acetate in isopropanol with 0.1% formic acid (mobile phase B) and 10 mM ammonium acetate in HPLC grade water with 0.1% formic acid (mobile phase C) was used. The column oven temperature was set to 45 °C and a flow rate of 500 μl/min was used. The analysis was assessed on a QTRAP 6500 (Sciex, Concord, Canada) equipped with a similar UHPLC system. The individual species were monitored in multiple reaction monitoring (MRM) mode in negative ion mode. 103 MRM transitions were monitored using a dwell time of 30 ms. Q1 and Q3 quadrupoles were operated in unit resolution mode. The collision energy was set at 80 eV for GM1s, 70 eV for GM2s, 60 eV for GM3s, 50 eV for GDs, and 40 eV for GQs and GTs. Nitrogen was used as collision gas. The ESI voltage was set at −4500 V and the ion source temperature at 400 °C.

#### Eicosanoid lipidomics

Eicosanoids were analyzed as described previously^24^. A similar instrument setup as for sphingolipidomics was used. The individual species were separated using a Phenomenex Jupiter, 250×2.0 mm column with a particle size of 5 μm (Phenomenex, Torrance, CA, USA). A 18 min gradient using water:acetonitrile:formic acid (63:37:0.02) (mobile phase A) and acetonitrile:isopropanol (50:50) (mobile phase B) was used. The column oven temperature was set to 60 °C and a flow rate of 300 μl/min was used. The analysis was assessed on a QTRAP 5500 (Sciex, Concord, Canada). The individual species were monitored in multiple reaction monitoring (MRM) mode in negative ion mode. 103 MRM transitions, split in two runs, were monitored using a dwell time of 15 ms. Q1 and Q3 quadrupoles were operated in unit resolution mode. The collision energy was set according to Deems et. al.^24^. Nitrogen was used as collision gas. The ESI voltage was set at −4500 V and the ion source temperature at 525 °C.

#### Lipid identification and quantification

The mass spectrometry data files were processed using LipidView V1.0.99 and MultiQuant 2.0 softwares to generate a list of lipid names and peak areas. Shotgun lipidomics data was processed in LipidView as described previously^30^. Briefly, the endogenous species were identified based on their characteristic fragment ions, neutral losses and parent ions. For instance, m/z 184.1, which is the characteristic headgroup ion of phosphatidylcholines (PC) and sphingomyelins (SM)^31^ was used to identify together with the parent mass the peaks observed in the mass spectrum of PIS 184.1 in positive ion mode. In a similar way the monitored acyl ions were utilized to identify the molecular species in negative ion mode^26^. For instance, identification of PC 16:0–18:1 requires corresponding signals from both the 16:0 (PIS of m/z 255.2) and the 18:1 (PIS of m/z 281.2) scans.

MRM data was processed in MultiQuant. The selected lipid characteristic ions and their parent masses in conjunction with retention time were used for the identification of the endogenous species. Information dependent acquisition (IDA) experiments were used for confirming identifications. MRM was used as survey scan to trigger the IDA, followed by enhanced product ion (EPI) scans of two most intense ions.

The identified lipids were quantified by normalizing against their respective internal standard and matrix type, e.g., volume for plasma, and presented accordingly (e.g., μM for plasma). Total lipid class concentrations were calculated by summing-up the concentrations of species belonging to the same class. Molar distribution percentages were generated by dividing all the observed lipid concentrations to the class total concentration within the sample.

Data filtering of the final data set was based on the frequency of individual lipid molecules observed throughout the collected data. Molecules observed from less than 75% of the samples, and molecules lacking lipid class specific internal standards were excluded. Molecules having four fold lower or higher concentration than the median of the group were considered as outliers and excluded. Filtering procedures were performed separately for both polarity modes and lipidomic assessment, prior to merging of the final lipidomic data set. All the calculations and data processing were performed using SAS 9.3 (SAS Institute).

Samples from 2 month and 3 month time points were analyzed separately from 7 month time point. Variation in instrument control samples between the batches were observed in ceramide (Cer, LacCer, Glc/GalCer and Gb3 lipid classes) and eicosanoid lipidomics platforms, and therefore correction factors were applied to endogenous lipid concentrations of these platforms.

Due to the use of correction factors, the ceramide and eicosanoid concentrations at 7 month time point are relative instead of absolute. Group comparisons within a particular time point are not affected by the use of correction factors.

The variation of endogenous lipid concentrations was due to internal standard (IS) issues (eicosanoids) and variation in total lipid extraction (ceramide species). The eicosanoid IS concentrations were varying between the two batches, which was observed from control samples. Variation was most likely due to evaporation of solvent from the IS vials, even though the vials were unopened and stored as recommended by the manufacturer.

The correction factors were calculated based on instrument control samples (ICs), which are included in all extractions to monitor quality of the lipidomics analysis. Pooled human plasma is extracted in ICs instead of a sample, concentrations of ISs are the same as in samples, and the extraction is done in the same fashion for ICs and samples. Ten replicate ICs were run per one sample set.

Endogenous lipid concentrations of eicosanoids and ceramide lipid species on 7 month time point samples were corrected by correction factors (k) obtained from the following equation. Peak area ratios (Endogenous area/IS area) from ten replicate IC runs were first calculated, and then averages of these ten ratios were used to determine the correction factors according to the equation. Obviously, the correction factors were determined only for those lipids that were detected in IC. For ceramide species the calculation was done per lipid extraction of the tissue type, for example ICs in lung sample batches were compared with each other.

To calculate corrected concentration, IS amount (pmol) in a sample was multiplied by k.

#### Quality control

To ascertain high quality data various controls were assessed. Data that fulfilled all the applied acceptance criteria were accepted. In all analyses instrument controls (IC), quality controls (QC), blanks and calibration lines were applied. ICs were based on fresh human plasma extracted (i.e., pooled extract) and analyzed in a similar way as the samples. The ICs served to monitor the performance and variation in the mass spectrometry analyses^19^. Dependent on the analysis and molecules (i.e., abundance) different thresholds were applied, but typical in the range of 20–50%. The samples were re-run if the thresholds were exceeded. QCs served in the same way as ICs, with the exceptions that the sample matrix was the same as the samples to be analyzed if available, and that they were individually extracted to include monitoring of the extraction efficiency. Slightly higher variation thresholds were typically applied for QCs. Blanks served to monitor the background noise and if the signal of a lipid molecule exceeded 25% in the blank it was excluded from the samples. Calibration lines served to monitor the linear response of the mass spectrometer. The analysis was accepted based on the linearity of the calibration lines. The linear regression must exceed 0.95 based on at least four out of six non-zero standards.

### Abbreviations

Lipids were annotated according to the described shorthand notation^32^ and LIPID MAPS nomenclature^33^. Briefly, the different lipid species of PC, phosphatidylethanolamine (PE), phosphatidylserine (PS), phosphatidylinositol (PI), phosphatidic acid (PA), phosphatidylglycerol (PG), and diacylglycerol (DAG) are listed with the two fatty acyl groups separated with a hyphen, e.g., PC 16:0–18:1, when positions are unknown and with a slash, e.g., PC 16:0/16:0, when known. LysoPC and lysoPE are abbreviated as LPC and LPE, respectively, and cholesteryl esters are abbreviated CE. Ether-linked phospholipids are shown as PC O (alkyl), PC P (alkenyl), PE O (alkyl), and PE P (alkenyl). Fatty acyl groups of ether-linked lipids and N-amidated fatty acyl groups for SM, ceramide (Cer), sphingolipids, and gangliosides are shown after the slash. Sphingolipids are: sphingosine (SPH), sphingosine-1-phosphate (S1P), sphinganine-1-phosphate (SA1P), glucosylceramide (GlcCer), lactosylceramide (LacCer), and globotriaosylceramide (Gb3). Gangliosides are: GD1, GM1, GM3, GQ1 and GT2. Fatty acids and eicosanoids are: docosahexaenoic acid (DHA), arachidonic acid (AA), eicosapentaenoic acid (EPA), 12-hydroxyeicosatetraenoic acid (12-HETE), 11-hydroxyeicosatetraenoic acid (11-HETE), 15-hydroxyeicosatetraenoic acid (15-HETE), 8-hydroxyeicosatetraenoic acid (8-HETE), 5-hydroxyeicosatetraenoic acid (5-HETE), prostaglandin E2 (PGE2), prostaglandin D2 (PGD2), 13-hydroxyoctadecadienoic acid (13-HODE), 9- hydroxyoctadecadienoic acid (9-HODE), 6-keto prostaglandin F1α (6-keto-PGF1alpha), prostaglandin F2α (PGF2alpha), tromboxane B2 (TXB2), tromboxane B3 (TXB3), 12-hydroxyeicosapentaenoic acid (12-HEPE), 14,15-dihydroxyeicosatrienoic acid (14_15-DHET), 11,12-dihydroxyeicosatrienoic acid (11_12-DHET), 8,9-dihydroxyeicosatrienoic acid (8_9-DHET), 5,6-dihydroxyeicosatrienoic acid (5_6-DHET), hydroxyoctadecatrienoic acid (13-HOTrE), 12-oxo-eicosatetraenoic acid (12-oxoETE), 5-oxo-eicosatetraenoic acid (5-oxoETE), 5-hydroxyicosapentaenoic acid (5-HEPE), 15-hydroxyicosapentaenoic acid (15-HEPE).

Data resulting from lipidomics analyses have been submitted to MetaboLights [Data Citation 3].

### Proteomics

#### Protein extraction

Tissue samples from the right lungs of six mice (i.e., six biological replicates) were analyzed for each exposure condition and time point. Lung tissue from months 1, 3, 5, and 7 for 3R4F and pMRTP exposure, and months 3, 5, and 7 for cessation and switch was available for quantitative proteomic analysis. The inferior lobe of the right lung was cryo-sliced into 40 μm thick slices (see above). Slices were homogenized in tissue lysis buffer (BioRad, Hercules, CA, USA) using the TissueLyser II bead-mill disruption system (Qiagen), and divided into two tubes. The first tube was used for two-dimensional (2D)-polyacrylamide gel electrophoresis (PAGE) analysis and the second for isobaric tags for relative and absolute quantitation (iTRAQ) analysis.

#### 2D-PAGE

Extracted proteins in lysis buffer were acetone-precipitated and resuspended in ReadyPrep 2D Starter kit rehydration/sample buffer (8 M urea, 2% 3-[(3-cholamidopropyl) dimethylamino]-1-propanesulfonate, 50 mM dithiothreitol, 0.2% Bio-lyte ampholytes pH gradient buffer pH 3–10, and bromophenol blue; BioRad). The protein concentration was determined using the modified Bradford protein assay (BioRad).

Isoelectric focusing was performed on 11 cm immobilized pH gradient strips (Readystrip IPG strip 3–10NL, BioRad) with a pI 3–10 nonlinear gradient, on an Ettan IPGphor 3 Isoelectric Focusing System (GE Healthcare, Little Chalfont, UK). Resuspended proteins were applied overnight to the strip to be rehydrated, then focused for 2 h at 150 V, 1 h at 500 V, 1 h at 1,000 V, then 4 h at 8,000 V to reach 25,000–30,000 V. After isoelectric focusing, the pH gradient strip was equilibrated with equilibration buffer 1 (7 M Urea, 75 mM Tris HCl (pH 8.8), 29.3% glycerol, 2% SDS, 1% dithiothreitol, and bromophenol blue) for 20 min, then alkylated with equilibration buffer 2, which was identical to buffer 1 but replaced 1% dithiothreitol with 2.5% iodoactemide.

The proteins were applied onto a Criterion XT Bis-Tris Gel, 12%, IPG+1 well, 11 cm IPG strip, 13.3×8.7 cm (W×L) (BioRad). Second-dimension separation was performed sequentially using a Criterion Dodeca Cell (BioRad). The separated spots were visualized using SYPRO Ruby staining (Invitrogen, Carlsbad, CA, USA). Stained 2D gels were scanned on an image scanner (Typhoon FLA 9500 scanner, GE Healthcare), and spot-intensity calibration, spot detection, background abstraction, and matching of 2D-PAGE were performed using SameSpots software (Nonlinear).

Spots of interest were cut from the gels, washed, and in-gel digested with 20 ng of Trypsin Gold, Mass Spectrometry Grade (Promega, Madison, WI, USA) using an ETTAN digester robot (GE Healthcare). Peptide mass fingerprint data and mass spectrometry spectra were acquired from matrix-assisted laser desorption/ionization time-of-flight mass spectrometry (UltraFleXtrem MALDI-TOF/TOF-MS; Bruker, Billerica, MA, USA). They were identified with the MASCOT search program and the Swissprot database.

Data resulting from proteomics 2D-PAGE analyses can be found in ‘Proteomics 2D gel ISA file (Metadata)’, ‘Proteomics 2D gel images’, and ‘2D-PAGE data—normalized matrix’ files [Data Citation 1].

#### iTRAQ

Extracted proteins in lysis buffer were precipitated using acetone, then resuspended in 0.5 M triethylammonium bicarbonate, 1 M urea, and 0.1% SDS (Sigma-Aldrich, St Louis, MO). The protein concentration was determined using the modified Bradford protein assay as mentioned above (BioRad). A total of 50 μg of protein was processed for iTRAQ 8-plex labeling according to the manufacturer’s instructions (AB Sciex, Framingham, MA, USA). Trypsin (Promega) was added to samples in a 1:10 trypsin:protein ratio (w/w) followed by overnight digestion at 37 °C. Samples were then labeled with the reporter-ion tags for different treatment groups. A common reference mix containing 50 μg of all protein extracts from each time point was also prepared and labeled with iTRAQ reporter-ion tag (iTRAQ channel 114). All labeled samples belonging to one iTRAQ set were pooled and dried in a SpeedVac. Samples were desalted using 1 cc C18 reversed phase Sep-Pak columns (Waters) according to the manufacturer’s instructions. For SDS-removal and to reduce the PEG carryover from the optimal cutting temperature embedding medium, 0.5 ml bed volume detergent removal columns were used (Pierce, Rockford, IL, USA). Samples were dried using the SpeedVac and resuspended in nanoLC buffer A (5% acetonitrile, 0.2% formic acid (both Sigma-Aldrich)).

Samples were analyzed using an EASY-nanoLC 1000 instrument connected online to a Q Exactive mass-analyzer (Thermo Scientific). Peptides were fractionated on a 50 cm C18RP RSLC EASY-spray column (2 μm particle size; Thermo Scientific) at a flow rate of 200 nl/min with a 200 min gradient from nanoLC buffer A (5% acetonitrile, 0.2% formic acid) to 40% acetonitrile, 0.2% formic acid. Each sample was injected twice with two different analysis methods: a fast and a sensitive method as described previously^34^. Both mass spectrometry runs were searched together against the mouse reference proteome set (Uniprot, version Aug_2013,) using Proteome Discoverer vers. 1.4.0.288 software (Thermo Scientific). Mascot (v.2.3, Matrixscience, Boston, MA) and SequestHT were used as search tools and resulting protein lists were merged. The Percolator node of the Proteome Discoverer software was used to estimate peptide-level adjusted *P*-values (q-values) and peptides were filtered for q-values <0.05 (i.e., the false discovery rate (FDR) was controlled at the 5% level). The quantification of iTRAQ reporter ions and the assignment of peptides to protein groups was performed using the Proteome Discoverer software. iTRAQ peptide-level quantification data were exported and further processed in the R statistical environment^35^, then filtered for ‘unique’ quantification results as defined by Proteome Discoverer software, e.g., removing redundant quantification results from multiple search engines. A global variance stabilizing normalization (VSN) was performed with the respective Bioconductor package in R^36,37^. Each iTRAQ reporter ion set was normalized to its median, and protein expression values were calculated as the median of these normalized peptide-level quantification values^38^.

Data resulting from proteomics iTRAQ analyses have been submitted to PRIDE [Data Citation 4].

#### Reverse phase protein array (RPPA)

The TissueLyser II bead-mill disruption system (Qiagen) was used for protein extraction from cryo-sliced right lung tissue samples. Protein extraction was performed according to the manufacturer’s instructions, using one 5 mm steel bead (Qiagen) and 400 μl CLB1 extraction buffer (Bayer Technology Services GmbH, Leverkusen, Germany). After extraction, the sample was centrifuged in a micro-centrifuge for 10 min at 16,000×g, and the cleared supernatant was transferred into fresh tubes for further analysis. Protein concentrations of collected supernatants were quantified using an EZQ test (Life Technologies). Protein extracts were stored at −80 °C until further use.

Analysis of protein extracts by Zeptosens reverse phase protein assay technology was performed as previously described^39^. Protein extracts were adjusted to uniform concentrations with CLB1 buffer (Bayer Technology Services GmbH). Adjusted extracts were further diluted with CLB1 buffer to a final spotting concentration of 0.1 μg/μl. Diluted extracts were printed at four serial dilutions (1.6-fold) onto Zeptosens hydrophobic chips (Bayer Technology Services GmbH) using a microarray printer (NanoPlotter 2.1, GeSiM, Grosserkmannsdorf, Germany). Each sample was processed with independent dilutions on separate arrays, i.e., eight spots per sample with four spots per array. Following array printing, arrays were blocked, washed, and dried according to the manufacturer’s specifications and stored at 4 °C in the dark until further use.

For the measurement of protein signals, arrays were incubated overnight at room temperature in primary antibodies at dilutions of 1:500 in CAB1 assay buffer (Bayer Technology Services GmbH). The following primary antibodies were used: anti-BRCA1 (sc646, Santa Cruz Biotechnology, Dallas, TX, USA), anti-C/EBPα (sc61, Santa Cruz Biotechnology), anti-C/EBPβ (sc150, Santa Cruz Biotechnology), anti-ITGB6 (sc15329, Santa Cruz Biotechnology), anti-PU.1 (LSC30679, LifeSpan BioSciences, Seattle, WA, USA), anti-SOCS3 (04004, Merck Millipore, Darmstadt, Germany), anti-STAT1 (9172, Cell Signaling Technologies, Danvers, MA, USA), and anti-STAT1Tyr701 (44376G, Life Technologies). Following washing, arrays were incubated for 2 h with either Alexa647- (Life Technologies) or Dylight650-labeled (Bio-Rad AbD Serotec GmbH, Puchheim, Germany) anti-species secondary antibodies, at dilutions of 1:500. After subsequent washing, stained arrays were imaged using an array scanner (ZeptoREADER, Bayer Technology Services GmbH), according to the manufacturer’s recommendations.

Data resulting from proteomics RPPA analyses can be found in ‘Proteomics reverse phase protein array ISA file (Metadata)’ and ‘Proteomics reverse phase protein array data’ files [Data Citation 1].

## Data Records

### Data record 1—Study overview file

The file ‘COPD2-Datadescriptor-Overview.xlsx’ gives for each sample the animal (with its coded animal number, CAN), the exposure group to which it belongs, the test or endpoint that was measured, and the name of the file where the data is given. [Data Citation 1].

### Data record 2—Moribund and dead animals and missing values

The file gives the description of moribund or dead animals which have been replaced by reserve animals in the study, as well as in the ‘Missing_Values’ tab the samples for which there is no available measurement in a particular endpoint with the associated reason. [Data Citation 1].

### Data record 3—Smoke chemistry

The file containing the concentrations of 58 harmful and potentially harmful constituents measured in aerosols from 3R4F and the pMRTP has been uploaded to Figshare [Data Citation 1]

### Data record 4—Body weight ISA file (Metadata)

The file ‘ISA_BodyWeight.xslx’ has been uploaded to Figshare [Data Citation 1]. It contains (i) general information on the experiment and the protocols related to exposure and body weight measurement on the ‘i_Organism’ tab, (ii) information on each animal in the ‘s_Organism’ tab, (iii) information on the body weight measurement in the ‘a_Organism_BodyWeight’.

### Data record 5—Body weight raw data

The file containing body weight measurements (Body_Weight_RawData.xlsx) has been uploaded to Figshare [Data Citation 1]. Briefly, it has the animal number information on the first row and the exposure group information in the second row. Then, the first column has the study day information, and each body weight recorded (in grams) is recorded in corresponding cells.

### Data record 6—Hematology ISA file (Metadata)

The file ‘ISA_Hematology.xlsx’ has been uploaded to Figshare [Data Citation 1]. It contains (i) general information on the experiment and the protocols related to the dissection and hematology measurement on the ‘i_Organism’ tab, (ii) information on each animal in the ‘s_Organism’ tab, (iii) information on the samples that were measured in the ‘s_DP_Hematology’ tab, and (iv) information on the measurements performed for hematology in the ‘a_DP_Hematology’ tab.

### Data record 7—Hematology raw data

The file ‘Hematology_RawData.xlsx’ contains hematology measurements and has been uploaded to Figshare [Data Citation 1]. Briefly, it has the animal number information on the first row and the exposure group information in the second row. Then, the first column has the name of the hematology parameter measured.

### Data record 8—Lung function ISA file (Metadata)

The file ‘ISA_Lung_Function.xlsx’ has been uploaded to Figshare [Data Citation 1]. It contains (i) general information on the experiment and the protocols related to the lung function measurements on the ‘i_Organism’ tab, (ii) information related to the exposure for each animal in the ‘s_Organism’ tab, (iii) information related to the animals for which lung function measurement were performed in the ‘s_DP_Lung_Function’ tab, and (iv) the links between the metadata and the measurements in the ‘a_DP_Lung_Function’ tab.

### Data record 9—Lung function raw data

Three consecutive perturbations were performed to record acceptable measurements (coefficient of determination >0.95) for each individual subject. FlexiVent software (SCIREQ) was used to analyze and calculate lung mechanics parameters. Measurements are made available as a data matrix ‘LungFunction_RawData.xlsx’ in Figshare [Data Citation 1]. Briefly, it has the animal number information on the first row and the exposure group information in the second row. Then, the first column has the name of the lung function parameter measured.

### Data record 10—Bronchoalveolar lavage fluid (BALF) ISA file (Metadata)

The file ‘ISA_BALF.xlsx’ has been uploaded to Figshare [Data Citation 1]. It contains (i) general information on the experiment and the protocols related to the BALF measurements on the ‘i_Organism’ tab, (ii) information related to the exposure for each animal in the ‘s_Organism’ tab, (iii) information related to the animals for which BALF measurement were performed in the ‘s_DP_BALF’ tab, and (iv) the links between the metadata and the measurements given in the Data records 11–13 in the ‘a_DP_BALF’ tab.

### Data record 11—BALF FACS analyses reports

The file ‘BALF_FACS.zip’ containing pdf reports of all BALF FACS analyses has been uploaded to Figshare [Data Citation 1].

### Data record 12—BALF free lung cell count and enzymatic activity

The file ‘BALF_INTERNAL_RawData.xlsx’ contains free lung cell counts and BALF enzymatic measurements and has been uploaded to Figshare [Data Citation 1]. Briefly, it has the animal number information on the first row and the exposure group information in the second row. Then, the first column has the name of the parameter measured.

### Data record 13—BALF analytes

Analyzed data are available in FigShare [Data Citation 1] in the file ‘BALF_MAP_RawData.xlsx’. For each analyte and each of the six batches of measurements, and in addition to the concentrations measured in the various supernatants, Myriad RBM provided the least detectable dose (LDD) and lower limit of quantification (LLOQ), which were not necessarily constant across the batches. The LDD was determined as the mean+3 standard deviations of 20 blank readings, while the LLOQ was the lowest concentration of an analyte that could be reliably detected in a sample and at which the total error met the laboratory requirements for accuracy. In this case, LLOQ was the concentration of an analyte at which the coefficient of variation of replicate standard samples was 30%. Given that these definitions of LDD and LLOQ are independent, sometimes LDD>LLOQ and vice versa.

In the original data reporting from Myriad RBM, not all measurements were reported as values. Some were instead reported as ‘quantity not sufficient for testing’ (QNS). Additionally, some myoglobin (only) measurements were reported as ‘>xxx’, where xxx represented the upper limit of quantification. Finally, for the first four shipments, some values were indicated as ‘<LOW>’, indicating that they could not be measured on the standard curve. Values below LDD or LLOQ were reported as values in these first four shipments. Myriad RBM reporting changed from shipment 5. ‘<LOW>’ values and values below LLOQ were reported as ‘<LLOQ’ for shipments 5 and 6. Moreover, if a measurement was <LDD but >LLOQ, it was reported as a value.

To accommodate these variations in reporting between shipments, the following decisions were made for our internal data analysis:

QNS values were considered to be missing.

Values ‘>xxx’ were substituted by 1.1 * xxx.

<LOW> or <LLOQ values were substituted by max(LDD, LLOQ)/2.

Values <LDD or LLOQ were substituted by max(LDD, LLOQ)/2.

Other values were left unchanged for data analysis.

### Data record 14—Histology and histomorphometry ISA file (Metadata)

The file ‘ISA_Histology_histomorphometry.xlsx’ has been uploaded to Figshare [Data Citation 1]. It contains (i) general information on the experiment and the protocols related to the dissection and histology and histomorphometry evaluations on the ‘i_Organism’ tab, (ii) information on each animal in the ‘s_Organism’ tab, (iii) information on the samples that were measured in the ‘s_DP_Histo’ tab, (iv) the links between the metadata and the histopathology measurements given in the Data records 15 in the ‘a_DP_Histopathology’ tab, (v) the links between the metadata and the histomorphometry measurements given in the Data records 15 in the ‘a_DP_Histomorphometry’ tab.

### Data record 15—Lung Histopathology and histomorphometry raw data

The file ‘Histo_Morpho_RawData.xlsx’ contains histopathology scores and histomorphometry measurements and has been uploaded to Figshare [Data Citation 1]. Briefly, it has the exposure group information on the first row, the time point information on the second row, and the animal number information in the third row. Then, the first column has the name of the finding evaluated, or the parameter in the histomorphometry measured and the second column the level at which this was done.

### Data record 16—Proteomics 2D gel ISA file (Metadata)

The file ‘ISA_prot2Dgel.xlsx’ has been uploaded to Figshare [Data Citation 1]. It contains (i) general information on the experiment and the protocols related to 2d gel proteomics on the ‘i_Organism’ tab, (ii) information on each animal in the ‘s_Organism’ tab, (iii) information on the samples that were measured in the ‘s_DP_Prot’ tab, (iv) the links between the metadata and the image files given in the Data records 17 in the ‘a_DP_Prot’ tab, (v) information on the data that was normalized in the ‘s_PR_Prot’ tab, (vi) the links between the metadata and the normalized matrix given in the Data records 18 in the ‘a_PR_Prot’ tab.

### Data record 17—Proteomics 2D gel images

The file ‘COPD2_2dgel_images.zip’ contains images of the 2d gels and has been uploaded to Figshare [Data Citation 1].

### Data record 18—2D-PAGE data—normalized matrix

Gel images (Data record 17) were analyzed using SameSpots software for the detection of spot volumes. Raw MALDI-TOF MS data were analyzed by BioTools software (Bruker) for protein identification using Uniprot databases.

Two quality control parameters were performed by the software. The first assessed the quality of gel images (saturation and intensity) and the second assessed the quality of the spots by aligning and comparing each gel with a mouse lung 2D-gel gold standard. The data from the lung tissues were normalized together, then experimental groups (Sham, 3R4F, pMRTP, Cessation, and Switching to pMRTP) were separately averaged and compared with each other. Spots with an ANOVA *P*-value <0.05 were selected and subsequently compared.

The file ‘COPD2_2dGEL_normalizedMatrix.xlsx’ was uploaded in Figshare [Data Citation 1].

### Data record 19—Proteomics reverse phase protein array ISA file (Metadata)

The file ‘ISA_protRPA.xlsx’ has been uploaded to Figshare [Data Citation 1]. It contains (i) general information on the experiment and the protocols related to reverse phase protein array proteomics on the ‘i_Organism’ tab, (ii) information on each animal in the ‘s_Organism’ tab, (iii) information on the samples that were measured in the ‘s_DP_Prot’ tab, (iv) the links between the metadata and the data given in the Data records 20 in the ‘a_DP_Prot’ tab.

### Data record 20—Proteomics reverse phase protein array data

For the correction of anti-species secondary antibody staining, arrays were assayed in the absence of primary antibodies. For the measurement of spotted protein, one blank chip (i.e., without antibody incubation) was stained with SYPRORuby. Scanned images were analyzed using ZeptoVIEW 3.1 software (Bayer Technology Services GmbH). Normalized fluorescence intensities (NFIs) for each sample and protein target were calculated as reference fluorescence intensities of primary antibody stained arrays (RFIprimary) corrected for secondary antibody staining (RFIsecondary), as well as relative spotted protein concentration (RFIprotein), determined by (RFIprimary—RFIsecondary)/RFIprotein, using ZeptoVIEW 3.1 software. NFI values were used for subsequent analysis.

A series of bovine serum albumin references were spotted on each array. Each sample underwent serial dilution (100, 75, 50, and 25%) to assess linearity. Prior to RPA analysis, all antibodies were validated by western blotting to assess their specificity.

The file ‘COPD2_RPA_RightLung.xlsx’ has been submitted to Figshare [Data Citation 1].

## Technical Validation

All equipment was validated prior to carrying out the study.

### Smoke generation validation

To characterize the test atmosphere and to check the reproducibility of MS generation and dilution, the following analytical parameters were determined at defined intervals: puff volume, TPM, CO, nicotine, formaldehyde, acetaldehyde, acrolein, particle size distribution, temperature, relative humidity, and flow rate through the exposure chamber (see Table 4).

The inlet temperature of the test atmosphere in each exposure chamber ranged from 22.1–23.7 °C.

### Animal traceability

The identity of the mice was traceable from the time of arrival to the time of dissection for each animal. Upon arrival, the number of mice transferred from the transport box to the cage was recorded. Mice were individually identified by subcutaneous transponder implantation such that each mouse had a unique transponder identity, coded animal number, and un-coded animal number.

### Animal welfare control

Body weight was measured biweekly to monitor normal growth. Additionally, before and after exposure, animals were visually inspected by trained staff and any irregularity addressed by the attending veterinarian. Access to food and water was checked regularly.

Hygiene was monitored within the animal holding and exposure rooms by carrying out microbial screening during the entire period of animal occupancy under the following schedule:

- Drinking water: screened every 2 weeks
- Diet: screened monthly
- Air: screened every 2 weeks
- Surface: screened weekly

The results of the above microbial screening procedures were consistently within specifications. Chamber pressure and air supply were monitored during the exposure period.

### Blinding and randomization

Random allocation of mice to experimental groups was conducted prior to exposure using a randomization sequence stratified by body weight. Blind testing was used for most endpoints; samples were identified by a coded animal number and the encoding was only revealed after the results were generated. Moreover, mice were randomized for necropsy, and samples were randomized (fully or by time point) for the generation of each endpoint whenever possible.

### Histology

At dissection time point 1, artefactual tissue alterations were observed in the lung parenchyma that biased the morphometrical analysis (measurement of mean chord length, destructive index, and bronchiolar attachments) such that a meaningful and comparative data evaluation was not feasible. These tissue alterations had been induced by mechanical compression of the lung parenchyma during tissue embedding in paraffin wax. Therefore, the first time point lacks morphometrical analysis. Moreover, in single animals at dissection time points 2, 3, and 4, an artefactual enlargement of the lung interstitium was seen particularly at the periphery of the main bronchus and the main pulmonary blood vessels. In some cases, the surrounding alveolar parenchyma was artificially compressed by interstitial thickening. This was attributed to the incorrect adjustment of the stretching table temperature, which was used to flatten tissue sections after sectioning at the microtome. Affected areas were identified histopathologically and excluded from morphometrical and histopathological evaluation; however, the frequency, distribution, and severity of the affected areas were not considered great enough to significantly affect data evaluation.

### Lung function measurements

The FlexiVent apparatus uses an indirect measurement technique that minimizes the measurement noise and calculates the amount of air delivered to the animal’s lungs from the movement of the ventilator’s piston. The result is an extremely accurate, reproducible measurement of the subject’s lung mechanics. The maneuvers performed are summarized in Table 5.

The effect of air compression in the ventilator compartment and resistance of the tubing had to be characterized before every experiment through a series of calibrations that are automatically taken into account in every animal measurement. At the start of each day of measurement, negative pressure calibration was performed with a ’two collected-point calibration’ (at approximatively −10 cm H_2_O and −90 cm H_2_O), followed by a flow channel calibration. Channel calibration was performed before each new experiment. To ensure maneuver reproducibility, scripts were prepared to describe those performed for each animal.

## Additional Information

**How to cite this article:** Ansari, S. *et al.* Comprehensive systems biology analysis of a 7-month cigarette smoke inhalation study in C57BL/6 mice. *Sci. Data* 3:150077 doi: 10.1038/sdata.2015.77 (2016).

## Supplementary Material

Supplementary Figure 1

Supplementary Figure 2

## Figures and Tables

**Table 1:** In-life observations

**Parameter**	**Method**	**Frequency**	**Number of Mice**	**Remarks**
Body weight	Gravimetry	Twice weekly	All	Individually
In-life observations	Non-systematic observation	Daily	All	—
	Group observations	Daily	All*	After exposure
	Individual observations according to checklist	Daily	One cage per exposure group	Immediately after removal from exposure chambers
Mortality	Observation	Daily	All mice	—

*With the exception of those mice selected for carboxyhemoglobin measurement or urine collection on the day of determination.

**Table 2:** Biomarkers of exposure—Carboxyhemoglobin (COHb) concentration (%), nicotine and cotinine concentrations in plasma (mean±s.e.m.)

**Parameter**	**Month**	**Sham**	**3R4F**	**pMRTP**	**Cessation**	**Switch**
COHb (%)	3	4.3±0.5	22.0±3.8	10.2±1.3	4.4±0.5	11.2±2.1
	4	4.9±0.6	28.3±5.3	10.6±0.8	5.6±0.5	13.2±1.1
	6	4.9±0.4	34.5±7.8	9.5±1.5	4.9±0.4	11.1±1.9
Nicotine (ng/ml) in plasma	7	1.6±0.5	6.4±0.5	3.9±0.4	1.7±0.6	7.1±2.1
Cotinine (ng/ml) in plasma	7	<LOD	35.7±6.4	31.1±4.4	<LOD	49.5±9.5
Trans-3'-Hydroxycotinine in urine (μmol/l)	6	<LOQ	102±9.8	75.7±9.4	<LOQ	60.0±5.3
Norcotinine in urine (μmol/l)	6	0.7±0.1	123± 17	123± 17	0.9±0.1	104±8.3
Cotinine in urine (μmol/l)	6	<LOQ	20.0±2.8	10.5±0.9	<LOQ	<LOQ
Nicotine-N'-oxide in urine (μmol/l)	6	<LOQ	39.3±6.8	39.3±6.8	<LOQ	29.1±3.0
Nornicotine in urine (μmol/l)	6	<LOQ	23.2±3.7	16.1±1.4	<LOQ	15.0±1.6
<LOD: below limit of detection, <LOQ: below limit of quantification.						

**Table 3:** Internal standards used for lipidomics analyses.

**Platform**	**Lipid class**	**Standard used**
Shotgun Lipidomics	PC	PC 17:0/17:0
	PE	PE 17:0/17:0
	PG	PG 17:0/17:0
	PS	PS 17:0/17:0
	PI	PI 17:0/17:0
	PA	PA 17:0/17:0
	PC-O/PC-P	PC 17:0/17:0
	PE-O/PE-P	PE 17:0/17:0
	LPL	LPC 17:0
	DAG	DAG 17:0/17:0
	TAG	TAG 17:0/17:0/17:0
	CE	D6-CE 18:0
	SM	SM (d18:1/12:0)
Sphingolipidomics	Cer	Cer(d17:1/18:0)
	GlcCer	D3-GlcCer(d18:1/16:0)
	LacCer	D3-LacCer(d18:1/16:0)
	Gb3	Gb3(d18:1/17:0)
	SPH	SPH d17:1
	S1P/SA1P	S1P d17:1
Gangliosides	GM1	D3-GM1-d18:1/18:0
	GM3	D3-GM3-d18:1/18:0
	GD1	D3-GM1-d18:1/18:0
	GT2	D3-GM1-d18:1/18:0
	GQ1	D3-GM1-d18:1/18:0
Eicosanoids	AA	AA-d8
	DHA	DHA-d5
	EPA	EPA-d5
	HETEs	5-HETE-d8/12-HETE-d8
	PGs	PGD2-d4
	HODEs	9-HODE-d4/13-HODE-d4
	TXBs	TXB2-d4
	HEPEs	12-HETE-d8
	DHETs	8,9-DHET-d11
	oxo-ETEs	5-HETE-d8/12-HETE-d8
	HOTrEs	9-HODE-d4

**Table 4:** Analytical characterization of the test atmosphere.

**Parameter**	**Assay Principle**	**Determination Schedule**
Total particulate matter	Gravimetry after trapping on Cambridge filters	During every exposure block
Carbon monoxide	Non-dispersive infrared photometry of gas/vapor phase	Continuously
Nicotine	Capillary gas chromatography after trapping on sulfuric acid-impregnated silica gel	During every exposure block, except during formaldehyde sampling
Formaldehyde, acetaldehyde, and acrolein	Reversed phase high-performance liquid chromatography of 2,4-dinitrophenylhydrazine (DNPH) derivatives after trapping in DNPH solution	≥once/week
Relative humidity	Capacitive	Continuously; sham group only
Temperature	Thermistor probe Pt100	Continuously
(Static) puff volume	Soap bubble flow meter	Before and after daily smoke generation
Flow rate through exposure chamber	Pressure difference over a Venturi tube	Continuously
Particle size distribution	PIXE impactor	Once

**Table 5:** Lung function measurements.

**Maneuver**		**Measurement**	**Unit**	**Interpretation cues**	
SnapShot perturbations (lung: single compartment)	Single frequency (150 breath/min) forced oscillation waveform (sinusoidal)	resistance (R)	cmH_2_O.s/ml	indicative of whole thorax	dynamic lung resistance
		compliance (C)	ml/cmH_2_O		ease with which lungs can be extended
		elastance (E)	cmH_2_O/ml		elastic rigidity of the lungs
Primewaves (lung: multiple compartments)	Broadband (multi-frequency) forced oscillation waveforms, typically denoted by duration (e.g., ‘Prime-8’) that also reflects frequency content	tissue elasticity (H)	cmH_2_O/ml/s	indicative of lung tissue	reflects energy conservation in the lungs
		tissue damping (resistance) (G)	cmH_2_O/ml/s		reflects energy dissipation in the lungs
		tissue hysteresivity	Î=G/H		
		Newtonian resistance (Rn)	cmH_2_O.s/	indicative of large airways	resistance of the central airways
			ml		
Pressure-volume loops	Slow (stepwise or continuous) inflation to total lung capacity (TLC) and deflation back to functional residual capacity	elasticity index in Salazar-Knowles equation (K)	/cmH_2_O		
		Maximum volume in Salazar-Knowles equation (A)	ml		indicative of total lung capacity
		quasi-static compliance (Cst)	ml/cmH_2_O		elastic recoil at given volume
		quasi-static elastance (Est)	cmH_2_O/ml		elastic recoil at given volume
		hysteresis (area in PV loops)	cmH_2_O/ml		measure of atelectasis
Positive end-expiratory pressure	Positive end-expiratory pressure of 2–3 cm H_2_O is adequate to maintain a normal end-expiratory lung volume in small animals	end-expiratory lung volume (Vtr end)	ml		
Negative pressure forced expiration	Lungs are inflated to TLC and then rapidly switched to a negative pressure reservoir, resulting in an expiratory flow	forced expiratory volume in 0.1 s (FEV0.1)	ml		
		forced expiratory volume in 0.2 s (FEV0.2)	ml		
		forced vital capacity (FVC)	ml		
		FEV0.1/FVC	%		
		FEV0.2/FVC	%		
