# Supplementary material for: Comprehensive systems biology analysis of a 7-month cigarette smoke inhalation study in C57BL/6 mice
Source: Sci Data. 2016 Jan 5;3:150077. doi: 10.1038/sdata.2015.77 (PMC4700839; doi:10.1038/sdata.2015.77)
Supplement: Supplementary Figure 1 [file sdata201577-s1.pdf]

Supplementary Figure 1

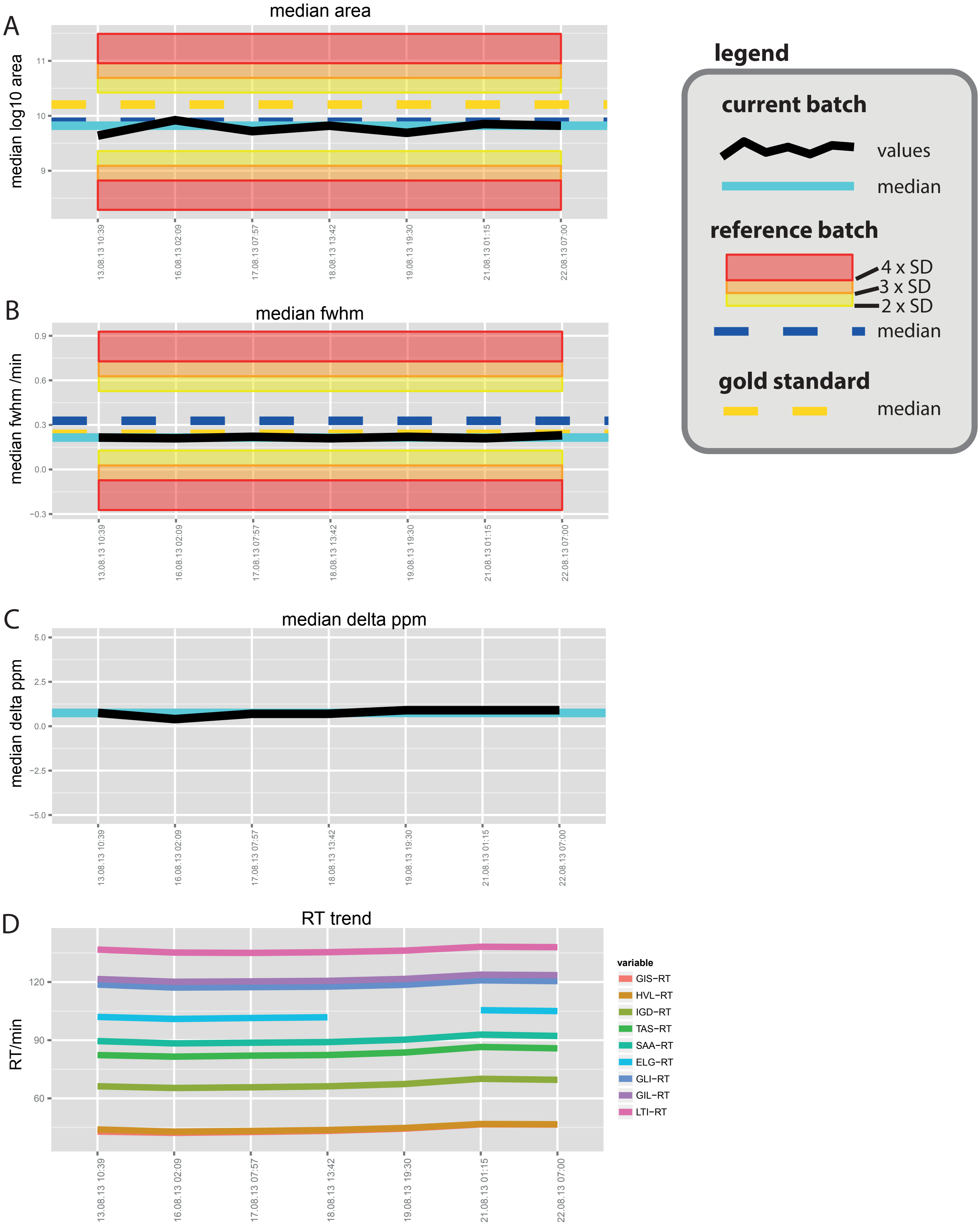

Performance evaluation of the Q Exactive mass-spectrometry instrument during analysis of the iTRAQ sample set. Before and during mass spectrometry analysis, the instrument performance was monitored with a 15-peptide standard mix (Thermo Scientific). (a) Median area of the peptide elution peaks. (b) Median FWHM of the elution peaks. (c) Median delta ppm. (d) Retention time stability. For a and b, the performance was compared with a "gold standard" set measured immediately after installation and operation qualification of the nanoLC and mass spectrometry instrument by the vendor. Values for the current batch were compared with the median and standard deviation (SD) limits of a well-performing reference batch. Note that the analysis is based on a subset of nine standard peptides that showed consistent and strong signals for the reference batch (indicated by their first three amino acids in d).
