# Supplementary material for: Comprehensive systems biology analysis of a 7-month cigarette smoke inhalation study in C57BL/6 mice
Source: Sci Data. 2016 Jan 5;3:150077. doi: 10.1038/sdata.2015.77 (PMC4700839; doi:10.1038/sdata.2015.77)
Supplement: Supplementary Figure 2 [file sdata201577-s2.pdf]

Supplementary Figure 2

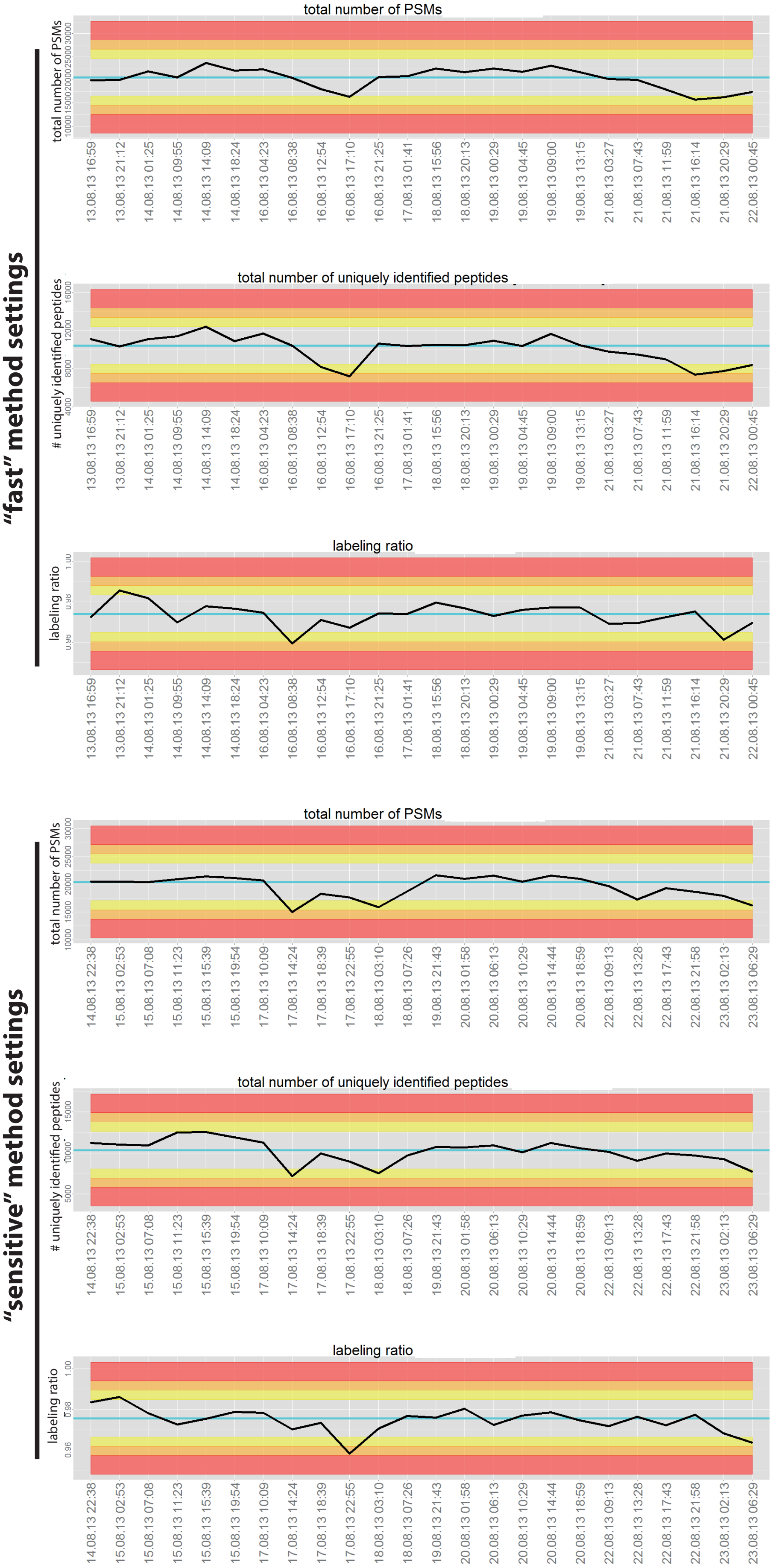

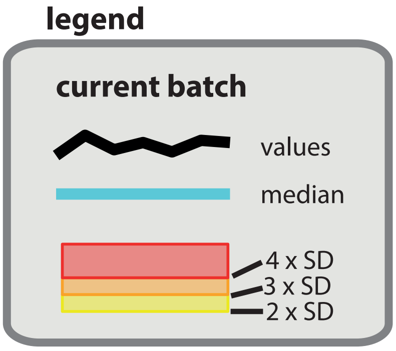

Quality evaluation of the multiplexed iTRAQ sample runs. Results for the "fast" and "sensitive" MS acquisition settings are shown. The MS runs were analyzed with a custom R-based QC pipeline based on OpenMS and ProteoWizard. For each acquisition setting, the number of peptide-to-spectrum matches (PSMs), the number of identified peptides, and the iTRAQ labeling efficiency are shown. The median and standard deviation (SD) limits of the analyzed sample batch are also indicated. The sample batch showed a consistently high iTRAQ labeling efficiency (>95%) and an overall consistent number of identifications.
